# Evaluation of factors that predict the success rate of trial of labor after the cesarean section

**DOI:** 10.1186/s12884-021-04004-z

**Published:** 2021-07-24

**Authors:** Yang Mi, Pengfei Qu, Na Guo, Ruimiao Bai, Jiayi Gao, Zhengfeei Ma, Yiping He, Caili Wang, Xiaoqin Luo

**Affiliations:** 1 Department of Obstetrics and Gynecology, Northwest Women’s and Children’s Hospital, Xi’an, 710061 China; 2 Translational Medicine Center, Northwest Women’s and Children’s Hospital, Xi’an , 710061 China; 3 Department of Nutrition and Food Safety, School of Public Health, Xi’an Jiaotong University, Xi’an , 710061 China; 4 Department of Health and Environmental Sciences, Xi’an Jiaotong-Liverpool University, Suzhou, 215123 China

**Keywords:** Vaginal birth after cesarean section, Trial of the labor after cesarean section, Intrapartum management, Prediction model

## Abstract

**Background:**

For most women who have had a previous cesarean section, vaginal birth after cesarean section (VBAC) is a reasonable and safe choice, but which will increase the risk of adverse outcomes such as uterine rupture. In order to reduce the risk, we evaluated the factors that may affect VBAC and and established a model for predicting the success rate of trial of the labor after cesarean section (TOLAC).

**Methods:**

All patients who gave birth at Northwest Women’s and Children’s Hospital from January 2016 to December 2018, had a history of cesarean section and voluntarily chose the TOLAC were recruited. Among them, 80% of the population was randomly assigned to the training set, while the remaining 20% were assigned to the external validation set. In the training set, univariate and multivariate logistic regression models were used to identify indicators related to successful TOLAC. A nomogram was constructed based on the results of multiple logistic regression analysis, and the selected variables included in the nomogram were used to predict the probability of successfully obtaining TOLAC. The area under the receiver operating characteristic curve was used to judge the predictive ability of the model.

**Results:**

A total of 778 pregnant women were included in this study. Among them, 595 (76.48%) successfully underwent TOLAC, whereas 183 (23.52%) failed and switched to cesarean section. In multi-factor logistic regression, parity = 1, pre-pregnancy BMI < 24 kg/m^2^, cervical score ≥ 5, a history of previous vaginal delivery and neonatal birthweight < 3300 g were associated with the success of TOLAC. The area under the receiver operating characteristic curve in the prediction and validation models was 0.815 (95% CI: 0.762–0.854) and 0.730 (95% CI: 0.652–0.808), respectively, indicating that the nomogram prediction model had medium discriminative power.

**Conclusion:**

The TOLAC was useful to reducing the cesarean section rate. Being primiparous, not overweight or obese, having a cervical score ≥ 5, a history of previous vaginal delivery or neonatal birthweight < 3300 g were protective indicators. In this study, the validated model had an approving predictive ability.

**Supplementary Information:**

The online version contains supplementary material available at 10.1186/s12884-021-04004-z.

## Background

Cesarean section is a common obstetric operation that can save the life of the mother and fetus. However, it can also cause some obstetric complications including maternal death, postpartum infection, uterine rupture, bladder injury, abnormal placenta, ectopic pregnancy, stillbirth, premature delivery and so on. In addition, cesarean section has been reported to be associated with changes in immune development, increased likelihood of allergies, late childhood obesity and asthma, and reduced diversity of gut microbiota [1].

Previously, the WHO expert group recommended a cesarean section rate of 10–15%, but the rate has risen sharply in most countries [2, 3]. Study has shown that once reaching 10%, further increases in cesarean section rate are unlikely to significantly reduce maternal, newborn and infant mortality [4]. One of the important factors leading to an overall increase of cesarean section rate is the repeated cesarean section for women with a history of previous cesarean section [5, 6].

Trial of labor after cesarean delivery (TOLAC) refers to a planned attempt to deliver vaginally by a woman who had a previous cesarean delivery, regardless of the outcomes. TOLAC provides women who desire a vaginal delivery the possibility of achieving vaginal birth after cesarean delivery (VBAC) [7]. For most women who had a cesarean section, VBAC is a reasonable and safe choice [8, 9]. In 2016, the introduction of the Second-child policy in China has led to an increase in the proportion of pregnant women who had cesarean section and second childbirth to the total delivery. However, currently, concerns about the risks of TOLAC have left only a small number of obstetricians and pregnant women to conduct TOLA C willingly. Therefore, effective measures need to be taken to help obstetricians predict and reduce the risks associated with TOLAC [10].

Our study conducted a retrospective analysis of the delivery methods of pregnant women who were pregnant again after cesarean section at the Northwest Women's and Children's Hospital from January 2016 to December 2018. We analyzed the factors that may affect the outcome of VBAC. In addition, we established a predictive model of TOLAC. This study can be used to strengthen the prenatal management and evaluation as well as to provide a theoretical basis for the construction of a vaginal trial risk prediction model after cesarean section.

## Methods

### Population and data sources

In this retrospective study, all the patients who delivered at Northwest Women’s and Children’s Hospital from January 2016 to December 2018 with a history of cesarean section and voluntarily selected TOLAC after cesarean section were recruited. The inclusion and exclusion criteria of the subjects were comprehensively referred to the VBAC guidelines developed by the Royal Academy of Obstetrics and Gynecology in the United Kingdom, Canada and the United States [7, 11, 12]. The TOLAC inclusion criteria were as follows: ① a history of cesarean section and a transverse incision of the lower uterus; ②  ≥ 37 gestation weeks and singleton live birth; ③ pelvis with normal shape and size; ④ the time of having the previous cesarean section was more than 2 years; ⑤ no contraindications to vaginal trial. Exclusion criteria were as follows: ① a history of uterine rupture during previous delivery; ② a history of two or more cesarean sections, or the last cesarean section was a classical one with a longitudinal incision of the uterus; ③ discontinuity of the anterior wall muscle layer of the lower uterine segment; ④ last cesarean section obstetric indications still existed; ⑤ suffering from other serious medical or obstetric complications.

### Perinatal management

Pregnant women were given full intrapartum management throughout their pregnancy including nutrition education and weight management. The delivery methods including the condition of pregnancy status, risk of childbirth for vaginal delivery and dissection were comprehensively evaluated and informed by at least 2 doctors with the position of deputy directors and above to the pregnant women and their families. Pregnant women were asked to provide a written informed consent prior to the study enrolment. If there was no indication of induction of labor, vaginal labor would be expected. If induction of labor was indicated, the method selected was according to the maturity of the cervix. During the process of vaginal trials, pregnant women were accompanied by an experienced midwife and fetal heart rate was continuously monitored for the preparation of cesarean section surgery at any time. If the delivery time exceeded 8 h, it should be reassessed whether the delivery was suitable for vaginal delivery, and patients would be transferred to cesarean section if necessary. If signs of abnormal fetal heart rate, threatened uterine rupture or uterine rupture were found, emergency cesarean section were implemented to deliver the fetus as soon as possible, and prepared for newborn resuscitation.

### Observation indicators

All clinical data of pregnant women were collected, including pregnant women’s age, education level, maternity times, pre-pregnancy body mass index (BMI), weight gain during pregnancy, scar thickness in the lower uterus, history of vaginal birth, number of antenatal visits, gestation week, Bishop’s score, time since last cesarean section and other maternal outcomes (including postpartum hemorrhage, transfusion rate, presence or absence of uterine rupture, puerperal infection, side injuries). In addition, neonatal outcomes (birth weight, APGAR score, and whether to transfer to the NICU) and other information were also obtained from the records.

### Statistical analysis

The SPSS ver.18.0 software was employed for statistical analysis. There were 80% of the included population who were randomly assigned to the training set, while the remaining 20% were assigned to the external validation set. Categorical variables were reported in frequency (percentage), and the difference between the groups was compared using the χ^2^ test or Fisher’s exact test. Continuous variables were analysed using a t-test or rank sum test.

In the training cohort, single-factor and multi-factor logistic regression models were used to determine factors related to successful TOLAC, and the correlation between related factors and successful TOLAC was expressed as an OR with 95% CI. The significant variables found in univariate analysis were further included in the stepwise multiple logistic regression model. The entry and exit criteria were *p* < 0.20 and *p* > 0.05, respectively. A nomogram was constructed based on the results of multiple logistic regression analysis, and the selected variables were included into the nomogram to predict the probability of successfully obtaining TOLAC. The area under the ROC curve (AUC) were used to judge the predictive ability of the model.

## Results

### Basic characteristics of participants

A total of 778 pregnant women were included in the study. The majority of them were younger than 35 years (71.34%), and only 73 pregnant women had a history of natural childbirth (9.38%). Among them, 595 pregnant women (76.48%) successfully received TOLAC, while 183 (23.52%) failed. The baseline characteristics of the training set (*N* = 551, 70.82%) and the validation set (*N* = 227, 29.18%) were shown in Table 1. No significant differences in the baseline characteristics were reported between the two groups.

**Table 1:** Participant’s characteristics of training set and validation set

Characteristics	Training set (*N* = 551)	Validation set (*N* = 227)	χ^2^	*P* value
Age (years)				
< 35	395 (71.69)	160 (70.48)	0.114	0.736
≥ 35	156 (28.31)	67 (29.52)		
Education level				
Lower than bachelor	293 (53.18)	128 (56.39)	0.668	0.414
Bachelor degree or above	258 (46.82)	99 (43.61)		
Gravidity				
2	220 (39.93)	89 (39.21)	0.035	0.852
> 2	331 (60.07)	138 (60.79)		
Parity				
1	241 (43.74)	104 (45.81)	0.281	0.596
> 1	310 (56.62)	123 (54.19)		
Pre-pregnancy BMI (kg/m^2^)				
< 24	428 (77.68)	171 (75.33)	0.500	0.480
≥ 24	123 (22.32)	56 (24.67)		
Excessive gestational weight gain				
No	376 (68.24)	164 (72.25)	1.216	0.270
Yes	175 (31.76)	63 (27.75)		
Lower uterine segment (LUS) thickness (mm)				
< 2.70	111 (20.15)	55 (24.23)	1.598	0.206
≥ 2.70	440 (79.85)	172 (75.77)		
Cervix Bishop score				
< 5	243 (44.10)	92 (40.63)	0.837	0.360
≥ 5	308 (55.90)	135 (59.47)		
Past vaginal delivery history				
No	498 (90.38)	207 (91.19)	0.124	0.725
Yes	53 (9.62)	20 (8.81)		
Time interval from previous cesarean section (year)				
< 9	479 (86.93)	197 (86.78)	0.003	0.955
≥ 9	72 (13.07)	30 (13.22)		
Neonatal birth weight (g)				
< 3300	208 (37.75)	88 (38.77)	0.071	0.791
≥ 3300	343 (62.25)	139 (61.23)		
Gestational age (week)				
< 39	173 (31.40)	75 (33.04)	0.200	0.655
≥ 39	378 (68.60)	152 (66.96)		

### Factors affecting the success of TOLAC

Table 2 showed the relationship between various single factors in the training set and the success of TOLAC. Pregnant women with a gravidity of 2, 1 parity, lower pre-pregnancy BMI (BMI < 24 kg/m^2^), Cervix Bishop score ≥ 5, a history of past vaginal delivery, longer interval from previous cesarean section (≥ 9 years), neonatal birth weight < 3300 g were more likely to succeed in TOLAC. In a further analysis, after multi-factor logistic regression, as shown in Table 3, parity = 1 (OR = 8.06, *p* < 0.001), pre-pregnancy BMI < 24 kg/m^2^ (OR = 2.40, *p* = 0.002), Cervix Bishop score ≥ 5 (OR = 3.46, *p* < 0.001), a history of past vaginal delivery (OR = 12.17, *p* < 0.001), and neonatal birth weight < 3300 g (OR = 4.44, *p* < 0.001) were closely related to successful TOLAC.

**Table 2:** Univariate logistic analysis of factors predicting successful TOLAC in the training set

Characteristics	Failure of TOLAC (*N* = 134)	Success of TOLAC (*N* = 417)	OR (95%CI)	*P* value
Age (year)				
< 35	88 (22.28)	307 (77.72)	1.46 (0.96, 2.22)	
≥ 35	46 (29.49)	110 (70.51)	1.00	0.076
Education level				
Lower than bachelor	80 (23.89)	223 (76.11)	1.05 (0.71, 1.55)	0.803
Bachelor degree or above	64 (24.81)	194 (75.19)	1.00	
Gravidity				
2	43 (19.55)	177 (80.45)	1.56 (1.03, 2.36)	**0.034**
> 2	91 (27.49)	240 (72.51)	1.00	
Parity				
1	31 (12.86)	210 (87.14)	3.37 (2.16, 5.26)	** < 0.001**
> 1	103 (32.23)	207 (66.77)	1.00	
Pre-pregnancy BMI (kg/m^2^)				
< 24	94 (21.96)	334 (78.04)	1.71 (1.10, 2.66)	**0.017**
≥ 24	40 (32.52)	83 (67.48)	1.00	
Excessive gestational weight gain				
No	83 (22.07)	293 (77.93)	1.45 (0.97, 2.18)	0.073
Yes	51 (29.14)	124 (70.86)	1.00	
Lower uterine segment (LUS) thickness (mm)				
< 2.70	20 (18.02)	91 (81.98)	1.59 (0.94, 2.70)	0.085
≥ 2.70	114 (25.91)	326 (74.09)	1.00	
Cervix Bishop score				
< 5	83 (34.16)	160 (65.84)	1.00	** < 0.001**
≥ 5	51 (16.56)	257 (83.44)	2.61 (1.75, 3.90)	
Past vaginal delivery history				
No	131 (26.31)	367 (73.69)	1.00	**0.001**
Yes	3 (5.66)	50 (94.34)	5.95 (1.82, 19.40)	
Time interval from previous cesarean section (year)				
< 9	125 (26.10)	354 (73.90)	1.00	**0.012**
≥ 9	9 (12.50)	63 (87.50)	2.47 (1.19, 5.12)	
Neonatal birth weight (g)				
< 3300	23 (11.06)	185 (88.94)	3.85 (2.36, 6.27)	** < 0.001**
≥ 3300	111 (32.36)	232 (67.64)	1.00	
Gestational age (week)				
< 39	37 (21.39)	136 (78.61)	1.27 (0.83, 1.95)	0.278
≥ 39	97 (25.66)	281 (74.39)	1.00

**Table 3:** Multivariate logistic analysis of factors predicting successful TOLAC in the training set

Characteristics	OR (95%CI)	*P* value
Parity (1 vs > 1)	8.06 (4.63, 14.01)	< 0.001
Pre-pregnancy BMI (< 24 vs ≥ 24)	2.40 (1.40, 4.14)	0.002
Cervix Bishop score (≥ 5 vs < 5)	3.46 (2.15, 5.56)	< 0.001
Past vaginal delivery history (Yes vs No)	12.17 (3.49, 42.48)	< 0.001
Neonatal birth weight (< 3300 g vs ≥ 3300 g)	4.44 (2.59, 7.62)	< 0.001

### TOLAC prediction model and model validation

**Fig. 1:**
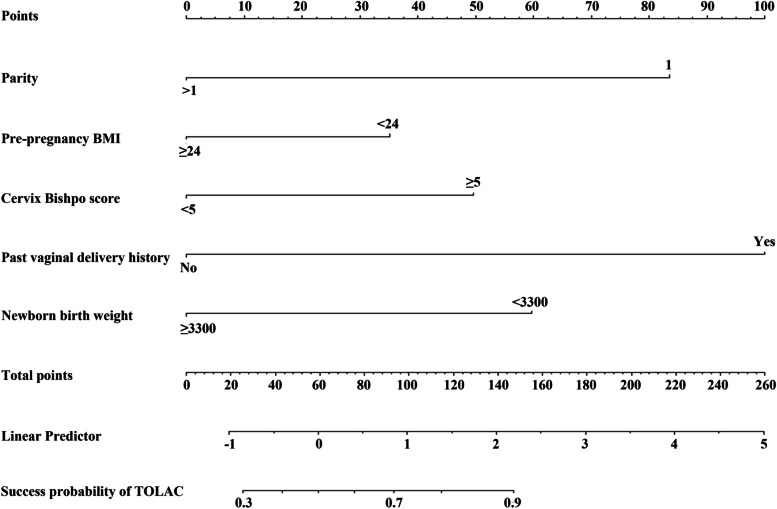
Nomogram for predicting success rate of TOLAC

**Fig. 2:**
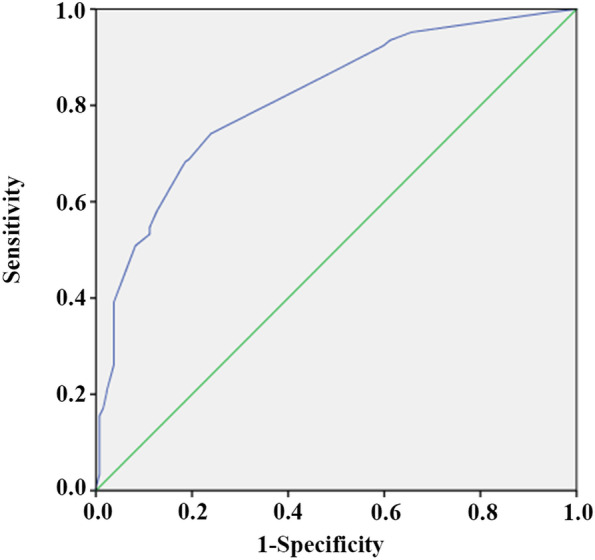
ROC curve in training set of women. The AUC in the prediction model was 0.815 (95% CI: 0.762–0.854). ROC, receiver operating characteristic curve; AUC, area under the ROC curve

**Fig. 3:**
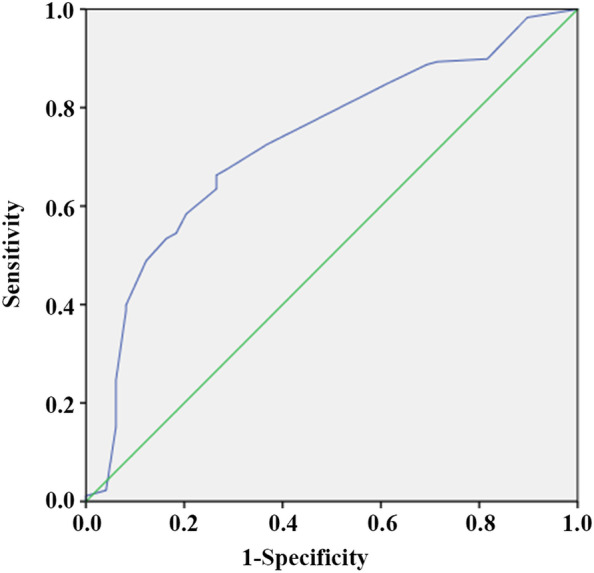
ROC curve in validation set of women. The AUC in the external validation model is 0.730 (95% CI: 0.652–0.808). ROC, receiver operating characteristic curve; AUC, area under the ROC curve

**Fig. 4:**
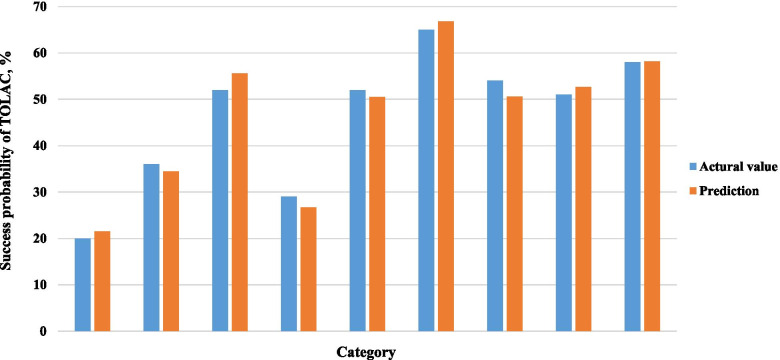
Success rate of the prediction model and the actual success rate calibration plot. Participants in the validation set were randomly divided into nine groups. Actual success rate is blue, and the predicted probability of success is orange

Based on the results of multi-factor logistic regression analysis, TOLAC's nomogram prediction model was obtained (Fig. 1). The AUC in the prediction model was 0.815 (95% CI: 0.762–0.854), and the AUC in the external validation model is 0.730 (95% CI: 0.652–0.808), indicating that the nomogram prediction model had medium discriminative power (Figs. 2 and 3 and Table S1). As shown in Fig. 4, participants in the validation set were randomly divided into nine groups. The TOLAC success rate predicted by the model was close to the actual TOLAC success rate, which proved that the model had a certain predictive ability.

### Comparison of pregnancy outcomes between VBAC group and TOLAC failure group

The outcome indicators of the TOLAC failure group and the success group were presented in Table 4. There were no significant differences in puerperal infection, NICU admission, and 5-min APGAR score between the two groups. The volume of blood loss during delivery and the incidence of blood transfusion during delivery in the VBAC group were significantly lower than those in the TOLAC failure group. The 1-min APGAR score was significantly higher in the VBAC group than the TOLAC failure group (*p* = 0.006). The neonatal birth weight in the VBAC group was significantly lower than the TOLAC failure group (*p* < 0.001).

**Table 4:** Comparison of pregnancy outcomes between VBAC group and TOLAC failure group

Characteristics	VBAC group (*n* = 595)	TOLAC failure group (*n* = 183)	t/Z/χ^2^	*P* value
Blood loss during delivery	253.39 ± 118.65	323.22 ± 169.71	-10.294	< 0.001*
Intrapartum blood transfusion	11 (1.85)	9 (4.92)	4.110	0.043
Puerperal infection	15 (2.52)	7 (3.83)	0.866	0.352
NICU admission	24 (4.03)	8 (4.37)	0.041	0.840
Apgar score (1 min)	8.95 ± 0.31	8.89 ± 0.37	-2.751	0.006*
Apgar score (5 min)	9.97 ± 0.17	9.96 ± 0.21	-1.287	0.198*
Neonatal birth weight	3331.32 ± 376.71	3477.87 ± 267.49	-5.346	< 0.001*

^*^ Rank sum test

## Discussion

Our study reported that the main factors affecting the success rate of TOLAC included parity, pre-pregnancy BMI, Cervix Bishop score, past vaginal delivery history and neonatal birth weight. These factors were of great value in predicting successful vaginal delivery. According to the logistic regression model, the nomogram model was constructed from these five factors. The AUC in the prediction and validation models was 0.815 (95% CI 0.762–0.854) and 0.730 (95% CI 0.652–0.808), respectively, which demonstrated that the nomogram prediction model had predictive value for successful vaginal delivery and a certain degree of accuracy.

A review reported that TOLAC is safe and feasible for most women with a history of cesarean section. The success rate of vaginal trial delivery is more than 75%, and the serious complications are less than 1% [13]. In a study conducted in Japan, there were 1532 women who tried TOLAC successfully gave birth with success rate of 88.6%, and 8 cases (0.46%) of uterine rupture [14]. Moreover, a very recent prospective study showed that a big proportion of patients (64.1%) chose TOLAC in Japan, and the final success rate was 91.3% with remarkably low uterine rupture rate (0.6%) [15]. The TOLAC success rate reported in our study was 76.48%, which was just moderate. When compared with the TOLAC failure group, the patients who successfully performed TOLAC had less blood loss and blood transfusion rate, and higher 1-min APGAR score. In general, TOLAC is potential strategy for decreasing the cesarean section rate and successful trials would reduce some important adverse outcomes.

Accordingly, one of the major concerns when conducting TOLAC is uterine rupture. A study has reported that the incidence of uterine rupture in patients undergoing TOLAC with a transverse incision in the lower uterus ranged from 0.5 to 0.9% [16]. It was generally suggested that the risk of uterine rupture is related to the thickness of the scar in the lower uterus. The continuity of the healing of the lower uterine myometrium has been proposed to more accurately predict whether the lower uterus will rupture. Since the current measurement of scar thickness and continuity standards are not uniform, the accuracy of B-ultrasound in evaluating the thickness and continuity of uterine scar myometrium is still controversial. In this study, the thickness of uterine scar was not significantly related to the success of TOLAC. The possible reason would be that if the thickness of the uterine scar of pregnant women was too low and not suitable for TOLAC, it would not be included in the study. Therefore, the thickness of the uterine scar was unlikely to be a factor affecting the outcome of TOLAC in our study. Other factors, such as advanced cervical opening, effacement, gravidity, parity, and prior vaginal delivery were also associated with successful vaginal birth [17]. In our study, the main factors related to the success of TOLAC were parity, pre-pregnancy BMI, cervical score, past vaginal delivery history, and neonatal birth weight.

The success rate of TOLAC is suggested closely related to the neonatal birth weight. Studies have confirmed that fetal weight is of high value in predicting TOLAC’s success [18, 19]. The larger the weight of the fetus, the lower the success rate of vaginal trial delivery. The reason may be that when the fetus is too heavy and the fetal head would be blocked, it may cause excessive traction of the lower uterine fibers, resulting in incomplete or complete rupture of the muscle layer of the lower uterus. This would then lead to failure of the vaginal trial. A previous study pointed out that compared to a neonate with a normal weight, the cesarean section rate of pregnant women with a baby weighing of more than 3450 g increased by 3 times, and the probability of VBAC was reduced by 50% for those with a neonatal weight of more than 3700 g [17]. Similarly, according to the results in our study, for pregnant women who are planning to undergo TOLAC, weight management can be carried out during pregnancy to keep the fetal weight within 3300 g.

Here, we found that the pre-pregnancy BMI of pregnant women in the TOLAC successful group was obviously smaller than that in the TOLAC failure group. After logistic regression analysis excluding the influence of confounding factors, pre-pregnancy BMI was still reported to had a significant impact on the success of TOLAC (OR = 2.40, 95% CI 1.40–4.14). Interestingly, there was no significant difference in weight gain during pregnancy between the two groups. Whether weight gain during pregnancy affects TOLAC is currently unclear. Previous study has provided evidence that weight gain during pregnancy and maternal BMI both associate with successful VBAC [20, 21]. Our results were consistent with a previous retrospective cohort study which emphasized that excessive weight gain during pregnancy was not a risk factor for failed TOLAC, even in obese patients [22]. Therefore, more studies are needed to confirm whether weight gain during pregnancy will affect the success rate of TOLAC.

A satisfactory prediction model could be clinically important to identify women with greater opportunities of a successful TOLAC. In a previous study, a nomogram model established had good performance at the high estimated probability of successful TOLAC for about 93% of women with an estimated ≥ 90% having a vaginal birth [21]. Although the probability in our study was relatively low, the TOLAC prediction model constructed here was useful to terminate the pregnancy in a timely manner, and to monitor closely during the labor process, which would increase the success rate of TOLAC and reduce the maternal and fetal complications related to cesarean section.

Some of the study limitations were that it was only a single-center study, and the scope of the survey was relatively narrow, which affected the extrapolation of the prediction model and may not reflect the actual situation of the Chinese population. Second, this study was a retrospective study, which will inevitably lead to the lack of some data analyses. In addition, the limited sample size may affect the extrapolation of the results of this study to a certain extent. Despite these limitations, there were a number of strengths associated with the study. First, this study conducted single-factor and multi-factor regression analysis on various factors that may affect TOLAC results, and identified several factors that had an impact on TOLAC results. By intervening these factors, the success rate of TOLAC can be improved clinically. In addition, a visual model has been constructed in our study, which can be tested in clinical practice. Also, this will provide a basis for future studies to explore the usefulness of the visual model in clinical practice.

## Conclusions

In conclusion, TOLAC is deemed a good choice for pregnant women to reduce unnecessary cesarean section. The predictive model has been demonstrated to more accurately predict the possibility of pregnant woman’s successful TOLAC, which further guarantees the safety of patients. In addition, pregnant women should improve pre-pregnancy or during-pregnancy management, such as good nutrition and weight management, will increase the success rate of TOLAC. Therefore, the results of our study have practical significance and can provide advice for clinicians to evaluate the eligibility of pregnant women who are planning to undergo TOLAC.

## Supplementary Information

**Additional file 1: Table S1.** The AUCs of the ROC curves for the nomogram and variables from the logistic regression model in the training set and validation set

## Data Availability

The datasets used and/or analysed during the current study are available from the corresponding author on reasonable request.

## Abbreviations

VBAC: Vaginal birth after cesarean section
TOLAC: Trial of the labor after cesarean section
ROC: Receiver operating characteristic curve
AUC: The area under the ROC curve
