# Supplementary material for: Evaluation of factors that predict the success rate of trial of labor after the cesarean section
Source: BMC Pregnancy Childbirth. 2021 Jul 24;21:527. doi: 10.1186/s12884-021-04004-z (PMC8305496; doi:10.1186/s12884-021-04004-z)
Supplement: Supplementary file 1 — Additional file 1: Table S1. The AUCs of the ROC curves for the nomogram and variables from the logistic regression model in the training set and validation set [file 12884_2021_4004_MOESM1_ESM.doc]

**Evaluation of factors that predict the success rate of trial of labor after the cesarean section**

Yang Mi^a^, Pengfei Qu^a^, Na Guo^a^, Ruimiao Bai^a^, Zhengfeei Ma^c^, Jiayi Gao^b^, Yiping He^a^, Caili Wang^a^, Xiaoqin Luo^b^*

^a^ Department of Obstetrics and Gynecology, Northwest Women’s and Children’s Hospital, Xi’an 710061, China

^b^ Department of Nutrition and Food Safety, School of Public Health, Xi’an Jiaotong University, Xi'an 710061, China

^c^ Department of Health and Environmental Sciences, Xi’an Jiaotong-Liverpool University, Suzhou 215123, China

*** Corresponding author**

Xiaoqin Luo, M.D.

Department of Nutrition and Food Safety, School of Public Health, Xi’an Jiaotong University, Xi'an 710061, China

Telephone: +86-29-82655111 Fax: +86-29-82655111

E-mail: [luoxiaoqin2012@mail.xjtu.edu.cn](mailto:luoxiaoqin2012@mail.xjtu.edu.cn)

**Table S1** The AUCs of the ROC curves for the nomogram and variables from the logistic regression model in the training set and validation set

|  | **Development group** | | **Validation group** | |
| --- | --- | --- | --- | --- |
|  | **AUC** | **95%CI** | **AUC** | **95%CI** |
| Nomogram variable | 0.815 | 0.762, 0.854 | 0.730 | 0.652, 0.808 |
| Parity | 0.636 | 0.593, 0.679 | NA | - |
| Prepregnancy BMI | 0.550 | 0.506, 0.593 | NA | - |
| Cervix Bishop score | 0.618 | 0.570, 0.665 | NA | - |
| Past vaginal delivery history | 0.549 | 0.529, 0.569 | NA | - |
| Neonatal birth weight | 0.636 | 0.596, 0.676 | NA | - |
